# A Case Series From a Multicentric Study: Can Artificial Intelligence (AI)-Enabled Chest X-Ray Assist in the Incidental Detection of Early-Stage Lung Cancers?

**DOI:** 10.7759/cureus.104881

**Published:** 2026-03-09

**Authors:** Deniz Koksal, Arunkumar Govindarajan, Azra Baykan, Gamze Durhan, Sevinc Sarinc, Meltem G Akpinar, Figen Demirkazik, Koushik M Mathivanan, Prasanna Vignesh, Santhosh Shivabasappa

**Affiliations:** 1 Chest Diseases, Hacettepe University, Ankara, TUR; 2 Radiology, Aarthi Scans, Chennai, IND; 3 Radiology, Hacettepe University, Ankara, TUR; 4 Pulmonology, Sri Ramachandra Medical College and Hospital, Chennai, IND; 5 Clinical Research, Qure.ai, Bangalore, IND

**Keywords:** artificial intelligence, chest x-ray, incidental pulmonary nodule, lung cancer, nodule

## Abstract

Lung cancer is the leading cause of cancer-related deaths worldwide. Early diagnosis is challenging, as patients are often asymptomatic. Low-dose computed tomography (LDCT) based screening has been shown to reduce mortality in high-risk individuals, but adoption is limited to a few countries. Chest X-ray is the most commonly used imaging modality in healthcare settings. Lung cancer can present as nodules on chest X-ray in the initial stages. Pulmonary nodules are often missed on routine chest X-rays. Artificial Intelligence (AI)-based chest X-ray software has shown promise in identifying nodules. We present a case series of five individuals in whom AI-enabled chest X-ray (qXR, Qure.ai, Mumbai, India) flagged suspicious pulmonary nodules on X-rays obtained for non-respiratory conditions. Computed tomography imaging and biopsy confirmed early-stage lung cancer in all five cases. Four individuals underwent curative surgical intervention with positive outcomes. One individual opted for chemotherapy alone. The use of AI software on routine chest X-rays identified suspicious nodules in asymptomatic individuals. The case series underscores the real-world impact of AI-enabled chest X-rays in the early detection of lung cancer.

## Introduction

Lung cancer is the leading cause of cancer-related deaths globally [1]. The majority of cases are diagnosed at advanced stages when curative surgical treatment is no longer feasible, resulting in poor survival outcomes. Screening with low-dose computed tomography (LDCT) in high-risk individuals has been shown to reduce mortality, but only a few countries have adopted formal screening programs [2,3]. Chest X-ray is a frequently used imaging test and is also performed as a part of annual health checks in many health systems. A pulmonary nodule, defined as a rounded or irregular opacity up to 3 cm in diameter on imaging, may represent benign disease or early-stage lung cancer. Pulmonary nodules are often overlooked on chest X-rays [4].

In recent times, artificial intelligence (AI)-based software has shown promise in detecting nodules on chest X-ray, and many commercial software programs are now available [5]. The qXR (developed by Qure.ai, Mumbai, India) is a deep learning based algorithm that can detect pulmonary nodules on frontal chest X-rays. The software device also categorizes the nodules as low or high-risk based on the size, calcification, border, and homogeneity of a nodule. The qXR tool has shown 85% sensitivity for nodule detection on chest X-rays [6]. However, most studies of these AI tools have used retrospective datasets, and prospective reports on patient outcomes remain limited.

The CREATE study (NCT05817110) is a prospective, multicentric study in which qXR-enabled interpretation was adopted for routine use on all chest X-rays across participating centers from April 2023 to December 2024. In this study, individuals flagged with a nodule by AI were assessed by radiologists and chest physicians, and nodules were evaluated further with a thoracic LDCT scan, as clinically indicated. The preliminary result from this study showed that an AI tool can reliably categorize incidental pulmonary nodules on chest X-ray as high- and low-risk of malignancy compared with radiologist assessment of LDCT [7]. We present a case series of five patients from this study who were diagnosed with early-stage lung cancer through the incidental detection of a high-risk pulmonary nodule by AI-enabled chest X-ray.

## Case presentation

**Figure 1:**
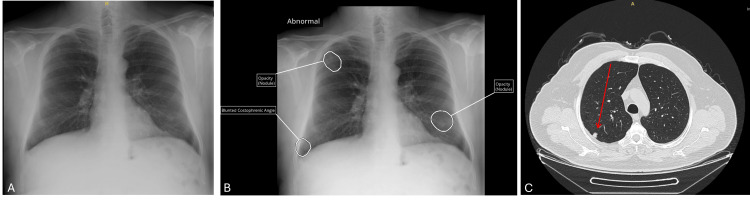
Chest X-ray and CT images of patient 1 A: Chest X-ray image without AI; B: Interpretation of chest X-ray with AI (suspicious nodules marked); C: Computed tomography (CT) image of the thorax (arrows show nodules)

**Figure 2:**
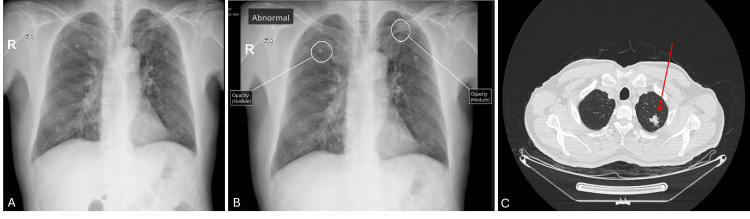
Chest X-ray and CT images of patient 2 A: Chest X-ray image without AI; B: Interpretation of chest X-ray with AI (suspicious nodules marked); C: Computed tomography (CT) image of the thorax (arrows show nodules)

**Figure 3:**
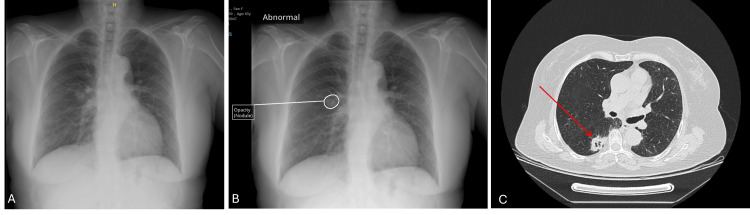
Chest X-ray and CT images of patient 3 A: Chest X-ray image without AI; B: Interpretation of chest X-ray with AI (suspicious nodules marked); C: Computed tomography (CT) image of the thorax (arrows show nodules)

**Figure 4:**
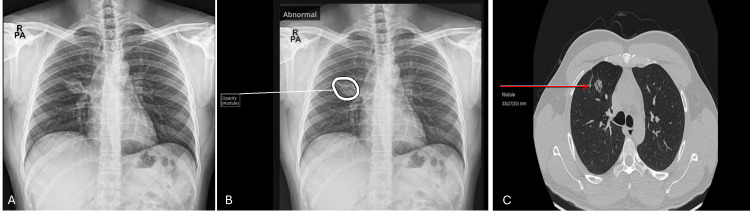
Chest X-ray and CT images of patient 4 A: Chest X-ray image without AI; B: Interpretation of chest X-ray with AI (suspicious nodules marked); C: Computed tomography (CT) image of the thorax (arrows show nodules)

**Figure 5:**
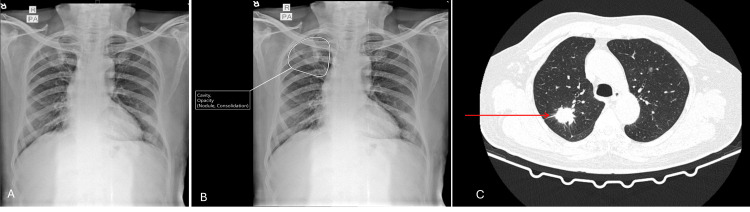
Chest X-ray and CT images of patient 5 A: Chest X-ray image without AI; B: Interpretation of chest X-ray with AI (suspicious nodules marked); C: Computed tomography (CT) image of the thorax (arrows show nodules)

All consecutive chest X-rays performed at participating centers were processed by the AI tool. Those flagged as high-risk were reviewed by a radiologist or pulmonologist, who determined whether further imaging was warranted. The five patients described here were identified through this prospective pathway; none had respiratory symptoms, and chest X-rays were obtained for unrelated clinical indications. The clinical presentation, nodule characteristics, biopsy details, and staging information are summarized in Table 1. Representative imaging of all cases is shown in Figures 1-5. In all five cases, the reviewing radiologist or pulmonologist agreed with the AI finding and recommended further imaging (LDCT ± PET-CT (positron emission tomography/computed tomography)). Two of the five patients had no history of smoking or any other recognized lung cancer risk factor.

**Table 1:** Summary of patients diagnosed with early-stage lung cancer through high-risk nodules detected by AI-enabled chest X-ray Lung-RADS: lung imaging reporting and data system, TNM staging: primary tumor (T), regional lymph node(s) involvement (N), and distant metastases (M), PET/CT: positron emission tomography/computed tomography, SUV: standardized uptake value, LDCT: low-dose computed tomography

Sl no	Clinical description	Smoking History	Lung RADS score	Biopsy	TNM stage	Intervention
							Case 1	A 57-year-old male with a known history of ulcerative colitis, chest X-ray screening obtained prior to anti-TNF treatment, an incidental nodule flagged by AI, and a PET-CT scan revealed a spiculated FDG-avid pulmonary nodule (9 × 10 mm) in the right upper lobe	Former, 18 pack years, quit 16 yrs ago	4X	Adenocarcinoma	Stage IB T2a(vpi)N0M0	Right upper lobectomy
Case 2	A 64-year-old male presented to the emergency room with fever and hyperglycemia, diagnosed with a urinary tract infection, an incidental nodule flagged by AI on chest X-ray, and a PET-CT scan revealed a 27 × 23 mm spiculated nodule in the left upper lobe with mild FDG uptake (SUVmax: 1.6)	Former, 45 pack-years, quit 3 yrs ago	4X	Adenocarcinoma	Stage IA2 pT1bN0M0	Left upper lobectomy
Case 3	A 65-year-old female presented to a family medicine physician for smoking cessation therapy, an incidental nodule flagged by AI on a chest X-ray, an LDCT identified a subpleural nodule in the right lower lobe, and a PET-CT revealed a nodule with a central cavity (33 mm), demonstrating increased FDG uptake (SUVmax: 11.4)	Current, 40 pack years	4X	Squamous cell carcinoma	Stage IIB pT3(satellite nodule)N0M0	Right pneumonectomy, Adjuvant three cycles of chemotherapy
Case 4	A 35-year-old male, asymptomatic, attended the annual health check. AI flagged an incidental nodule on chest X-ray, LDCT scan revealed a soft tissue density (2.2*2.8*2.7) with spiculated margin in the right upper lobe, PET scan revealed FDG-avid (SUV max 9.3) spiculated nodule (2.6*2.7*2.0 cm) in the anterior segment of the right upper lobe	No	4B	Adenocarcinoma	Stage IIB pT1cN1M0	Right upper lobectomy, adjuvant three cycles of chemotherapy
Case 5	A 75-year-old male, asymptomatic, attended the annual health check. AI flagged an actionable nodule on chest X-ray, LDCT scan revealed an inhomogeneous nodule (2.6*2.5*2.7) with spiculated margin in the posterior segment of the right upper lobe. PET-CT scan showed an FDG-avid heterogeneously enhancing nodule (2.8*2.9*4.2 cm) with a SUV max of 5.39 with spiculated margins in the posterior segment of the right upper lobe	No	4B	Adenocarcinoma	Stage IIA T2bN0M0	Three cycles of chemotherapy; the patient declined surgery

## Discussion

This case series highlights the role of AI-enabled chest X-rays in identifying potentially malignant pulmonary nodules on X-rays obtained for non-respiratory conditions. In all five cases, AI flagging enabled timely referral for further imaging (LDCT/PET-CT) and subsequent diagnosis of early-stage lung cancer. Without the assistance of AI, these cases would likely have been missed or delayed.

The lung cancer incidence continues to rise in the general population, including younger, non-smoking groups [8,9]. The absence of an official lung cancer screening program remains a significant challenge in many countries, and most lung cancers are diagnosed at advanced stages. Detecting early is critical, as surgical intervention for early-stage disease offers survival rates that exceed 90% [10]. Two of the five patients in this series had no traditional risk factors (age or smoking history), underscoring the limitations of risk-factor-based screening criteria.

Chest X-rays are performed routinely and are readily available in most health facilities. In many instances, nodules are missed or not reported on chest X-ray [4,11]. Solitary pulmonary nodules are an initial finding in 20-30% of lung cancer cases [12]. Employing AI for all routine chest X-rays provides an opportunity to detect incidental pulmonary nodules in a broad, unselected population. In a two-year study, Kwak et al. found that using AI to interpret routine chest X-rays led to the unexpected detection of 13 cases of resectable early-stage lung cancer [13]. Both Kwak et al. and Hwang et al. also reported that AI detected clinically significant incidental nodules on chest X-rays performed in non-respiratory outpatient clinics [14].

The qXR AI tool described here also categorizes the nodules as high or low risk, which can help clinicians make an informed decision based on clinical context. LDCT and incidental chest X-ray lung nodule programs can be complementary and can expand access to early lung cancer detection to different risk groups [15]. AI-enabled chest X-ray analysis is particularly valuable in settings with limited radiologist availability or high imaging workloads.

The cost-effectiveness of integrating AI into routine chest X-ray interpretation is an important consideration for widespread implementation. Formal economic analyses are needed before routine deployment can be recommended; however, early evidence suggests that the incremental cost of AI-assisted review may be offset by the downstream savings associated with stage-shift toward earlier, more treatable disease [16]. These questions warrant further research.

This case series has several important limitations. First, it is a small descriptive series and does not capture the overall diagnostic accuracy of the AI chest X-ray algorithm. The final results of the ongoing CREATE study will throw light on the clinical yield of lung cancer and performance metrics of AI-enabled chest X-ray, including data on the total number of chest X-rays processed, the number flagged as high-risk, and the number confirmed by biopsies. Second, there is inherent potential for false-positive flagging with AI, and radiologist confirmation is necessary before undertaking an extensive diagnostic workup. Third, as all five patients were asymptomatic and chest X-rays were obtained for non-respiratory reasons, this series is proof of concept for AI-assisted incidental detection rather than evidence for large-scale implementation. Further prospective research is required to evaluate the broader impact of routine AI-enabled chest X-ray on lung cancer diagnoses, and to determine the optimal integration of this approach alongside established LDCT screening programs.

## Conclusions

The use of artificial intelligence-enabled chest X-rays can identify potentially suspicious nodules on X-rays obtained for non-respiratory conditions. Many settings lack structured screening programs, and AI-enabled chest X-rays can facilitate the early detection of lung cancer.
